# Progress and Challenges in the Process of Using Solid Waste as a Catalyst for Biodiesel Synthesis

**DOI:** 10.3390/molecules30153243

**Published:** 2025-08-01

**Authors:** Zhaolin Dong, Kaili Dong, Haotian Li, Liangyi Zhang, Yitong Wang

**Affiliations:** College of Metallurgy and Energy, North China University of Science and Technology, 21 Bohai Street, Tangshan 063210, China; dzl200109@163.com (Z.D.); 17739439899@163.com (K.D.); 18032815354@163.com (H.L.); 15530326449@163.com (L.Z.)

**Keywords:** solid waste, catalyst, esterification, transesterification, biodiesel

## Abstract

Biodiesel, as one of the alternatives to fossil fuels, faces significant challenges in large-scale industrial production due to its high production costs. In addition to raw material costs, catalyst costs are also a critical factor that cannot be overlooked. This review summarizes various methods for preparing biodiesel catalysts from solid waste. These methods not only enhance the utilization rate of waste but also reduce the production costs and environmental impact of biodiesel. Finally, the limitations of waste-based catalysts and future research directions are discussed. Research indicates that solid waste can serve as a catalyst carrier or active material for biodiesel production. Methods such as high-temperature calcination, impregnation, and coprecipitation facilitate structural modifications to the catalyst and the formation of active sites. The doping of metal ions not only alters the catalyst’s acid-base properties but also forms stable metal bonds with functional groups on the carrier, thereby maintaining catalyst stability. The application of microwave-assisted and ultrasound-assisted methods reduces reaction parameters, making biodiesel production more economical and sustainable. Overall, this study provides a scientific basis for the reuse of solid waste and ecological protection, emphasizes the development potential of waste-based catalysts in biodiesel production, and offers unique insights for innovation in this field, thereby accelerating the commercialization of biodiesel.

## 1. Introductory

With the development of the global economy, under the influence of industrialization and urbanization, energy demand has become a focal point [1]. According to the World Oil Outlook (WOO) 2024 released by oil-exporting countries, global oil demand is expected to reach 120.1 million barrels per day in 2050 [2]. Among them, India and China will see the largest increase in demand of 8 million barrels per day and 2.5 million barrels per day, respectively. The shortage of fossil fuels and the resulting environmental pollution are important factors limiting global development. Therefore, finding a sustainable fuel that can replace oil is the main research direction. Biomass energy has attracted more and more attention due to its renewability, raw material richness, and short-term production. Biodiesel, as a typical ‘green energy’, has the potential to provide sustainable development of the economy and environment [3].

Biodiesel (FAME) was the term given to fatty acid methyl esters or fatty acid ethyl esters that were formed through the process of esterification or transesterification of vegetable oils (such as rapeseed oil, soybean oil, peanut oil, corn oil, cottonseed oil, etc.), animal oils (such as fish oil, lard, butter, lamb oil, etc.), waste oils or microbial oils, under the action of catalysts [4]. Biodiesel combustion can reduce net CO_2_ emissions by 78%, CO emissions by 46.75%, and particulate matter emissions by 66.7% [5]. Biodiesel has similar physical and chemical properties to traditional diesel, such as a higher cetane number, suitable viscosity and density, and lower cloud point and pour point. Biodiesel can be directly used or mixed with traditional diesel for diesel engines, and used in agriculture, industry, and other fields [6,7]. In order to improve the production efficiency of biodiesel, the use of catalysts has become an indispensable part. In recent years, many types of catalysts have been found, including homogeneous catalysts, heterogeneous catalysts, and bioenzyme catalysts [8,9]. Homogeneous catalysts have the advantages of a high reaction rate and mild reaction conditions [10]. However, alkaline catalysts were prone to saponification reaction during the reaction process, and acidic catalysts have a high reaction temperature. Both of them were corrosive to the reaction equipment and cannot be recycled, which can easily cause waste and the pollution of resources [11]. The heterogeneous catalyst has strong recyclability and no corrosivity, However, its disadvantages include slow diffusion-limited reaction speed, a relatively complex preparation process, and high preparation cost [12]. The applicable reaction conditions of biological enzyme catalysts were relatively mild, environmentally friendly, and highly active, but the loss of enzyme activity and the long reaction time limited their development [13]. Therefore, developing novel catalysts from solid waste for the production of sustainable and economical biodiesel is a prerequisite for industrialization.

According to the ‘2024 Global Waste Management Outlook’ jointly released by UNEP and the International Solid Waste Association, the direct cost of global waste management in 2020 was USD 252 billion [14]. If the secondary pollution caused by improper waste disposal increases the cost to USD 361 billion, in 2050, waste management costs are projected to be USD 640.3 billion. If a large amount of waste is not properly disposed of, it is easy to produce groundwater pollution, soil pollution, and harmful gas pollution only through dumping and landfills, and this can also affect human health through the food chain [15]. The current waste management system contributes to 5% of the global anthropogenic greenhouse gas emissions. If effective measures are not taken, it is expected to reach 2.38 billion metric tons of CO_2_ equivalent by 2050 [16]. In the context of developing a circular economy, various regions of the world explored a green and efficient solid waste treatment method [17]. These solid wastes contain organic or inorganic substances. Through calcination, impregnation, and other activation techniques, researchers prepare solid wastes into carriers or catalysts. This will help to reduce the total amount of solid waste, improve the environmental remediation capacity, and reduce the production cost of sustainable energy. It is a solid waste utilization method with development potential [18]. Currently, there are studies related to the production of biodiesel using catalysts prepared from waste from various industries. They achieved a high biodiesel yield and cycle efficiency. Their commercial potential can also be demonstrated through cost and environmental analysis.

This paper provides a comprehensive overview of organic and inorganic solid wastes used as catalysts for biodiesel synthesis. The latest innovations in the design, synthesis technology, and application of different waste-based catalysts were summarized to confirm their usefulness. The economic and technical feasibility and environmental impact of waste-based catalysts were also discussed, and their economic and environmental benefits were emphasized. Finally, the future development trends and challenges were put forward, which provides a reference for the study of solid waste catalysts. Compared with other related review articles, this article covered a variety of solid waste types and comprehensive preparation methods. It will provide readers with more detailed references as well as a favorable theoretical basis for the commercialization of solid waste for the catalyst preparation of biodiesel.

## 2. Organic Solid Waste-Derived Catalysts

### 2.1. Biomass Organic Solid Waste

Biomass organic solid waste is the waste produced by humans through using plants, animals, and microorganisms. This waste is potentially threatening to the environment, climate, water, soil, and so on. However, this waste is also an important renewable resource. At present, the application of biomass organic waste is mainly the material utilization and energy recovery. As a rich carbon source, it is also commonly used for the preparation of biodiesel catalysts.

#### 2.1.1. Plant Solid Waste

Plant waste consists of organic material from plants that has lost most or all of its value and often cannot be easily recycled or reused in its current form. Plant waste contains a large amount of C, O, and metal elements, which can be used as an excellent raw material for the production of biodiesel catalysts. The production of catalysts from plant wastes not only solves the problem of waste management, but also promotes the development of biodiesel preparation technology.

##### Agricultural Waste

Agricultural wastes mainly include plant fiber wastes such as straw, residual plants, weeds, deciduous leaves, fruit shells, vines, branches, and other wastes. Table 1 summarizes the preparation of catalysts from different agricultural wastes for the production of biodiesel. Eriola Betiku et al. [19] used sandbox seed shells calcined at 500 °C in a muffle furnace to obtain catalysts for sandbox oil-catalyzed biodiesel. The ash content of the calcined sandbox seed shell was confirmed by EDS analysis. The content of C and O elements decreased, and the content of the K element increased from 0.52% to 44.99%. K can prevent the leaching of metal elements in biomass ash and improve its reusability in esterification and transesterification reactions. Under optimal conditions, the highest biodiesel yield of 98.26 wt% was predicted by modeling. It was obviously higher than the use of mature and immature plantain mixtures to prepare catalysts for biodiesel production [20], especially in the transesterification reaction using a microwave-assisted method to reduce the reaction time greatly. For some agricultural wastes, different catalysts can be prepared from different parts of them. Bidangshri Basumatary et al. [21] prepared catalysts by the open burning and calcination at 550 °C of different parts of banana fruits. FESEM confirmed that the morphology of the calcined catalyst was sponge-like. The surface was rougher than that of the open burning catalyst. The content of K in different parts of the banana increased after calcination, which raised the pH and alkalinity of the catalyst. The yield of Jatropha oil catalyzed by calcined banana peel was 98 ± 0.27%. Although this yield is lower than the 99.4% achieved with the KOH-impregnated nanotube catalyst [22], the reaction conditions are mild, allowing the production of high-quality biodiesel at lower energy costs. In addition to using a single waste, catalysts can be prepared by mixing multiple wastes. Orange peel and banana peel powder at different calcination temperatures were mixed in different proportions to catalyze linseed oil [23]. It can be confirmed by FI-IR and XPS that elemental Ca was the most abundant in the catalyst and all of it has been present in the form of CaO. It has the advantage of increasing alkalinity and decreasing solubility. However, CaO easily absorbs water in the air and causes denaturation. Its storage method will also affect the performance of the catalyst.

Although the catalyst prepared by calcining agricultural waste can catalyze a higher biodiesel yield, the treatment of the catalyst with acid or alkali helped to change the structure of the catalyst to improve the reusability of the catalyst. Lidya Novita et al. [32] mixed palm kernel shell ash, SiO_2_, and NaOH in the solid phase and used catalysts prepared by a mechanical method. It can be clearly observed from the SEM image that the 450 °C catalyst has a well-ordered structure with a uniform particle size and obvious grain boundaries. However, after six times during which it reacted, the pores of the catalyst were blocked by other reactants. The biodiesel yield decreased from 99.01% to 80.15%. In another study, corn pod powder was treated with hydrochloric acid and reacted with papaya seed oil to obtain a biodiesel yield of 98.94% for up to 10 cycles [33]. When using the same raw material to prepare sulfonated carbon catalyst, the average biodiesel yield over five cycles (88 ± 0.9%) was significantly lower than that of the hydrochloric acid-treated catalyst [36]. The acid leaching process of corn pods led to many wider openings on the surface of the catalyst. The positive electrostatic repulsion between the surface of the catalyst and the reactant confirmed the stability of the catalyst in the transesterification reaction. Heterogeneous catalysts were prepared by dissolving various metal oxides in H_2_SO_4_, H_3_PO_4_, HCl, and then mixing with coffee waste [37]. H_2_SO_4_ and H_3_PO_4_ can dissolve TiO_2_, and the average pore size of Ti(SO_4_)_2_ was the largest, as confirmed by XRD and XRF. The proportion of strong acid sites was significantly increased by H_2_SO_4_ treatment, especially the intermediate and strong acid sites, which facilitate proton transfer during esterification. The esterification efficiency of different sulfonated catalysts decreased in the following order: Ti(SO_4_)_2_ > Zr(SO_4_)_2_ > Al_2_(SO_4_)_3_ > MgSO_4_ > non-catalytic. Ti and Zr can adjust the hydrophobicity of the surface, promote the esterification reaction, and reduce the formation of by-products.

**Figure 1:**
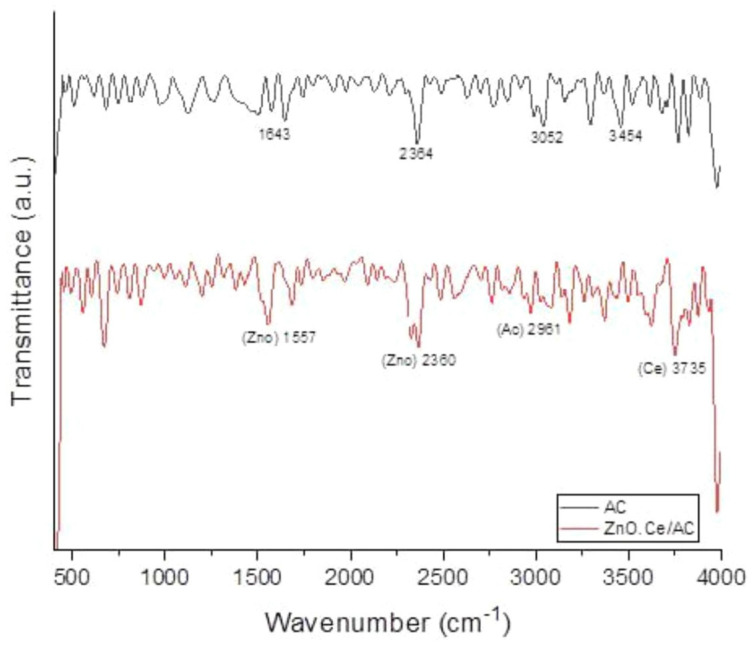
FTIR analysis for AC and ZnO. Ce/AC. Reproduced with permission from Reference [40].

The use of the microwave-assisted method in the transesterification process for the synthesis of biodiesel eliminates the wall effect in heat transfer. This in turn reduces the total energy consumption [38]. The alkaline catalyst was obtained by calcining the pods at different temperatures in a muffle furnace. EDX analysis showed that the highest content of alkaline metal was produced at 500 °C [39]. Moderately basic sites at 357 °C and 419 °C were found in CO_2_-TPD, promoting deprotonation reactions of methanol. Under the framework of RSM, the central composite rotation design (CCD) was used to optimize the relevant process parameters, and variance analysis was used to fit the established model. When the microwave power was 600 W, the molar ratio of methanol to oil was 9.87:1, the catalyst loading was 1.00 wt%, the reaction time was 3.03 min, and the maximum yield of biodiesel was 98.41%. In another study, a bifunctional catalyst was synthesized using avocado seeds. Under optimal conditions (100 °C, 3 wt% catalyst loading, and 500 W microwave power), the biodiesel yield was 99.6% [40]. The absorption peaks of Zn-O and Ce-O bonds were found in the FI-IR spectra, which confirmed the existence of ZnO and CeO_2_ (Figure 1). ZnO provided basicity and thermal stability, and CeO_2_ enhanced redox properties. The combined effect of the two improved the catalytic potential of the catalyst. The biodiesel yield of the hydrothermally synthesized and sulfonated core–shell catalyst under microwave assistance was 96.73% [41]. The complex preparation process increased the production cost of the catalyst. Considering the economy of biodiesel production, it is a potential development direction to select solid waste-based catalysts to prepare biodiesel by the microwave-assisted method.

##### Forestry Waste

Forestry waste refers to the waste generated in the process of forestry production, forest renewal, and forest product processing. It includes logging residues, clearing forest tending residues, and wood processing residues. The combination of nanotechnology and forestry waste are used to prepare green nanocatalysts. The metal bonds and synthetic gravity between nanoparticles lead to nanoparticle aggregation. The combination of nanotechnology and forestry waste are used to prepare green nanocatalysts. Because the combination of covalent bonds and metal bonds is difficult to destroy, it is easy to form a stable framework [42]. Plant extracts can also prevent the excessive aggregation of particles, resulting in more dispersed nanoparticles, making the surface of the catalyst rougher. The size change in the catalyst leads to a higher specific surface area, which is more conducive to the adhesion of the catalytic active material. Under the optimal conditions, the yield of biodiesel is 94%, and the high catalytic performance is maintained after 5 cycles [43]. In another study, the same method was used to prepare nanocatalysts by adding different forestry waste extracts to FeSO_4_ [44] and Sb_2_(SO_4_)_3_ [45] solutions. Plant extracts can be used as a reducing agent and stabilizer in hydrothermal synthesis [46]. The doping of different metal ions not only improves the thermal stability of the catalyst, but also shows a granular shape, uniform distribution, and some slight aggregation. In the GC-MS analysis of the catalyzed biodiesel, it was found that most of it was present as unsaturated fatty acids. This reduced the reaction of peroxidation and improved its physicochemical properties. High-temperature calcination can reshape the crystal form. Due to the fast reaction, the crystal growth time is short. Therefore, some active substances are attached to the surface of the carrier in the form of small crystals, which have a large specific surface area.

Hydrothermal carbonization is a new technology for the preparation of carbon-based catalysts [47]. In this process, a mixture of biomass material and water reacted to produce a hydrocarbon structure containing a large number of oxygen-containing functional groups [48]. Aliyu et al. used hydrothermal carbonization technology on palm leaves to prepare catalysts for catalyzing palmitic acid fat distillates. Higher oxygen content in the catalyst, confirmed by EDX analysis, was linked to other atoms in covalent bonds to form compounds [49]. Lignin decomposition was accomplished between 1067.70 °C and 1271.29 °C with good thermal stability. After H_3_PO_4_ activation and H_2_SO_4_ activation, the catalyst reached a higher surface area of 110.73 m^2^/g and a biodiesel yield of 93.5%. This result was significantly different from the specific surface area of 12.40 m^2^/g and the biodiesel yield of 90.4% reported by Lokman I.M. et al. [50]. In another study, a magnetic catalyst was prepared by the hydrothermal reaction of Jatropha shell and Fe_3_O_4_ nanoparticles, then activated using H_2_SO_4_ [51]. H_2_SO_4_ sulfonation not only increased the total acidity of the catalyst by 1680%, but also produced a larger particle size. After 5 cycles of the catalyst, the S content decreased from 3.62 wt% to 1.62 wt%. Therefore, we hypothesize that the decrease in catalytic efficiency was related to the sulfonic acid group. In the magnetic catalyst prepared by double hydrothermal precipitation, pyrolysis, and sulfonation, the sulfonation of H_2_SO_4_ increased the total acid density of the catalyst by more than 10 times, and the carbon layer on the surface was more tightly bound to the sulfonic acid group [52].

Brindhadevi et al. [53] prepared biodiesel by the photocatalytic method. The catalysts were first prepared by mixing *Pongamia pinnata* leaf extract and titanium isopropoxide, followed by precipitation with alkali and finally calcination. TiO_2_ can inhibit ultraviolet absorption and inhibit excitation, and SiO_2_ has a good support effect and adsorption effect. It was found by SEM that TiO_2_ was inserted in the SiO_2_ interstitials. There were agglomerations of TiO_2_ in some of the regions. This resulted in the specific surface area of TiO_2_ and SiO_2_ composites being smaller than that of SiO_2_. The highest biodiesel conversion was 97.5% and 98.6% using photocatalytic versus conventional transesterification, respectively. Although the conversion rate of the photocatalytic method was lower than that of the conventional transesterification method, it provided a more environmentally friendly method for the preparation of biodiesel.

#### 2.1.2. Animal Solid Waste

Animal waste contains a large number of hydrocarbon organic substances, which are widely distributed and easy to recycle. Biodiesel catalysts can be prepared using physical or chemical treatments, which can be a potential resource for the recycling and utilization of organic waste.

Fish scale is a shell that protects itself from water loss. As a kind of animal derivative, fish scale is rich in HAP (hydroxyapatite), accounting for about 46% of the total mass. HAP can be converted into β-tricalcium phosphate (β-TCP) by high-temperature calcination. R. Chakraborty et al. [54] developed a fish scale-based catalyst and explored the effect of different calcination temperatures on the catalyst. After TGA and XRD analysis, the catalyst mass was 52% of the sample mass at a calcination temperature of 1000 °C. The peak corresponding to the active β-Ca was enhanced, showing the overall structural stability of the fish scale-based catalyst. β-Ca_3_(PO_4_)_2_ acts as the active component of the catalyst, promoting the conversion of methanol molecules into methoxy groups. The preparation of biodiesel using calcium methanol as a catalyst also involves the desorption of O^−^ from calcium methanol to convert methanol into methoxy groups, following the same reaction mechanism [55]. Based on the RSM model of the FCCD, the optimal reaction conditions were determined. A maximum biodiesel yield of 97.73% was obtained, which was nearly equal to the yield of biodiesel catalyzed by calcium methoxide. The application of the flash evaporation process not only improved the efficiency, but also reduced the cost. It replaces the traditional calcination process to treat the fish scale, which can completely decompose the fish scale into a hydroxyapatite precursor. After sulfonation with concentrated sulfuric acid, the atomic percentage of Ca decreased to 17.07%, and the atomic percentage of O increased to 64.84%. The S atom replaced the P atom, and the hydroxyapatite was converted into calcium sulfate [56]. It was found in SEM that the catalyst had smaller and irregularly shaped porous particles. The sulfonation process led to an increase in surface area and provided more active sites. Finally, the dilution of the sulfate and leaching of the active sites resulted in a lower conversion of the catalyst under the third cycle. Soraida Bosoy et al. [57] developed the preparation of new magnetic catalysts by the mechanical mixing of three solids: iron hydroxide, high-temperature calcined fish scales, and magnetic NiFe-LDH hydrolyzed by urea using the hydrothermal reaction. In the SEM image the composite catalyst presented three different shapes of particles: flake, spherical, and small pores. The composite catalyst had a high surface area of 383 m^2^/g, which is significantly higher than the 24.05 m^2^/g surface area of zeolite-like catalysts prepared using the coprecipitation method [58]. The magnetic properties of the catalyst were verified by the M−H curve. It was confirmed that it was a superparamagnetic material with high saturation magnetic strength, low coercivity, and narrow remanence. After five cycles, the saturation magnetism decreased slightly, which facilitated the recovery and utilization of the catalyst.

Animal feces refer to the waste discharged from the digestive system through the anus or cloaca of animals. Chicken manure contains a large amount of calcium compounds. Maneerung T. et al. [59] dried chicken manure and calcined it at 550–950 °C to prepare an active calcium oxide catalyst. The calcination at higher temperatures increases the crystal form of CaO and causes agglomeration. SEM images showed that with the increase in temperature, the catalyst particles were agglomerated. This led to an increase in particle size, produced more CaO phases and added more basic sites. The basicity of the catalyst was determined by the Hammett index. Especially at 850 °C, the total basicity of the catalyst was up to 12 mmol/g and reached 90% biodiesel yield. The solid carbon waste in pig manure (SM) was used as a biochar-based catalyst for the transesterification of waste edible oil by pyrolysis technology [60]. The catalyst with 650 °C pyrolysis temperature was more mature as it had a higher dehydrogenation rate. This can increase the degree of carbonization to form a porous media structure with 96.67% biodiesel yield. The CaO/ZrO_2_ catalyst prepared by the impregnation method exhibits mesoporous and macroporous categories. Although the mass transfer process is promoted, the biodiesel yield was 95.9% [61]. Maafa I.M. et al. [62] prepared CaO catalysts by co-precipitation using solutions obtained from shrimp feces by demineralization, depotassiumization, and deacetylation. Through N_2_ adsorption–desorption experiments, the surface area of CPW-CaO was calculated to be 26.8 m^2^/g. The calcination of the catalyst removed the organic matter, resulting in a larger surface area and pore size. It was used to produce biodiesel from soybean deodorizer distillate, and a yield of 97% was obtained under optimal conditions. SDDO biodiesel was produced in accordance with the EN 14214:2010 standard [63] in all its characteristics. The sulfur content was less than 0.3 parts per million, which can reduce SO_2_ emissions.

#### 2.1.3. Microbial Solid Waste

Microorganisms are a group of organisms, including bacteria, viruses, fungi, protozoa, and a few algae. Among them, algae are the raw material of the third generation of biodiesel. They are not only rich in lipids but also contain organic matter such as protein, carbohydrate, and cellulose. The preparation of solid catalysts from algae for the preparation of biodiesel not only broadens the utilization of algae but also reduces the wasting of resources. Farobie et al. [64] studied the effect of different calcination temperatures on the catalyst. BET analysis showed that the surface area and pore size of the catalyst calcined at 600 °C were 20.87 m^2^/g and 12.41 nm, respectively, compared with those calcined at 500 °C and 700 °C. This increases the catalytic reaction rate. After impregnation with a KOH solution, more alkaline sites were attached to the surface of the catalyst. This promoted the deprotonation and conversion of methanol, and the highest biodiesel yield was 93%. In another study, a carbon-based magnetic solid acid catalyst prepared from microalgae residue was used for transesterification to produce microalgae biodiesel [65]. The internal structure of microalgae was destroyed after lipid extraction, which was not conducive to the activation of the catalyst. The pore-forming agents (NaCl, KOH, CuCl_2_) were used to enlarge the internal pores of the microalgae. This provided reaction paths for reaction molecules and reduced the influence of diffusion resistance. When CuCl was used as a pore-forming agent, the magnetic group replaced the free Cu^2+^ to generate Fe^3+^, which reduced the magnetism of the catalyst and had the lowest porosity. The basicity of KOH was not conducive to the adsorption of the sulfonic acid group diagram. The use of NaCl can not only increase the acidity but also increase the specific surface area of the catalyst. The metal–organic framework (Cu-BTC) had an ideal pore structure. It was used with an algae residue synthesis catalyst for the production of biodiesel from algae lipid [66]. The surface of Cu-BTC with a bipyramidal structure contains a large number of oxygen-containing functional groups after it is combined with algae residue biochar. The transesterification reaction was promoted, resulting in a biodiesel yield of 92.56%. The characteristics of macroalgae biodiesel were in line with ASTM D6751-24 specification [67]. Its cloud point and pour point are 2.5 °C and −1.9 °C, respectively. Compared with traditional diesel, it has low temperature resistance, which was also due to the oxygen-containing substances in algae biodiesel.

**Figure 2:**
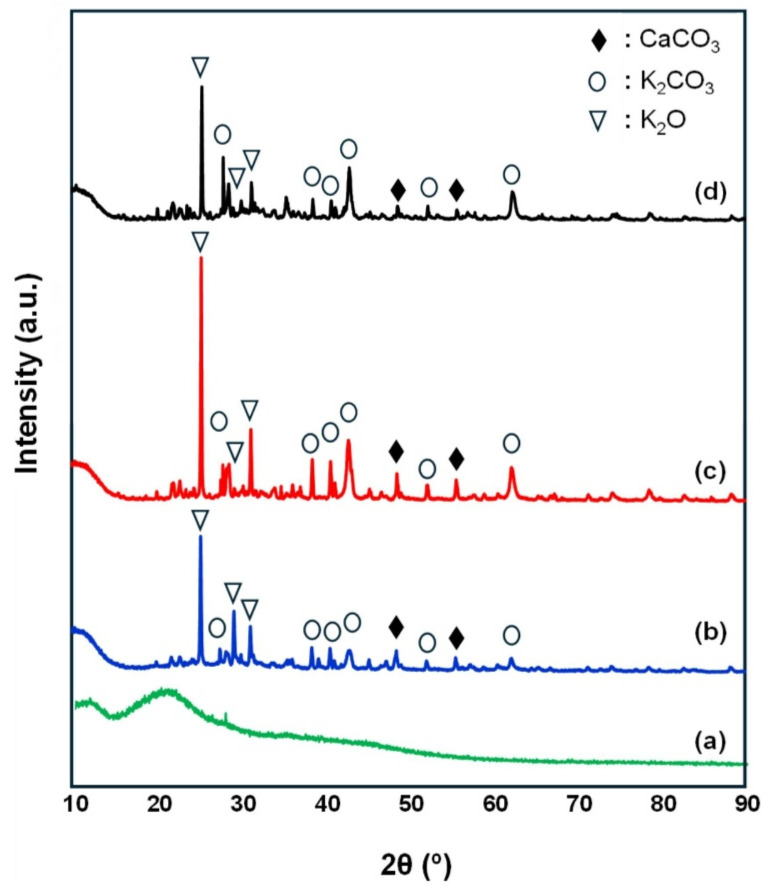
(**a**) XRD patterns of raw U. lactuca and calcined U. lactuca at (**b**) 500, (**c**) 600, and (**d**) 700 °C. Reproduced with permission from Reference [70].

The use of algae mixed with other solid waste to prepare catalysts not only improves the catalytic effect but also reduces the cost of the catalyst. Sargassum-calcined biochar and eggshell-calcined CaO were mixed and added to a K_2_CO_3_ solution to prepare a catalyst for catalyzing edible oil [68]. AFM showed that the microstructure of the catalyst was CaO embedded in the biochar cavity, and K_2_CO_3_ adhered to the surface of the structure to form a rough surface. Based on SEM observation, the stalagmite-like particles were eroded, the biochar texture disappeared, and the catalyst cracked after multiple cycles. This was mainly due to the adsorption of high acid value oil by the catalyst and the chemical reaction between the catalyst and the substances (H_2_O and CO_2_) in the air to destroy its structure [69]. The green algae *Ulva lactuca* were dried and ground, calcined at different temperatures, and then impregnated with a KOH solution to prepare a catalyst for the production of biodiesel from waste edible oil [70]. The XRD spectrum showed that when the calcination temperature increases from 500 °C to 600 °C, K_2_CO_3_ decomposes into more active K_2_O to increase the alkalinity of the material (Figure 2). When the calcination temperature is 700 °C, the catalyst particles sinter, the crystal structure is destroyed, and the crystallinity decreases. Using the microwave-assisted method, although the reaction time was 25 min, the biodiesel yield was up to 92% [71]. The catalytic biodiesel flash point was lower than the ASTM D6751-24 standard [67], which posed a certain storage and transportation risk. In the later stage, it was necessary to focus on the improvement of the flash point.

### 2.2. Industrial Organic Solid Waste

Solid wastes containing organic substances discharged from industrial (pharmaceutical and chemical, fine chemical) production are called industrial organic solid wastes. Due to their complex composition and poor biodegradability, their treatment has historically not always been proven. However, they are used as a catalyst not only to realize the effective utilization of solid waste but also serve to reduce environmental pollution. Pectin is a biopolymer. The ALP-n (n is the calcination temperature) catalyst was prepared by the co-precipitation method. Following the incorporation of pectin, the content of sodium aluminate in the catalyst increased. Nevertheless, elevated temperatures have been demonstrated to encourage the development of aluminum hydroxide, a process that is incompatible with the nucleophilic reaction involving methanol [72]. Therefore, attention should be paid to the calcination temperature in the production of catalysts. Waste bleached soil is the industrial waste of refined vegetable oil, which is characterized by a composition of 20% organic matter and 80% inorganic matter. It was used as support for the catalyst and then impregnated with KOH for activation [73]. This provided additional sites for physical adsorption and chemical adsorption in the catalytic process and promoted the reaction of water molecules with active surface sites. Concurrently, the doping of KOH resulted in the destruction of the catalyst structure, showed a scaly layer, the slit-like pores were loaded with activated substances, and the maximum biodiesel yield was 80.26%. Although the efficiency was not high, it also showed potential for a treatment method involving waste bleached soil. Ion exchange resins consist of solid plastic particles of organic polymer chains containing charged functional groups used to produce deionized water. The catalysts were prepared by loading a strong base anion exchange resin onto polystyrene and then activated with NaOH to catalyze animal fats and waste cooking oils. Three transesterification methods were employed in the preparation of biodiesel. It was found that the highest yield of biodiesel prepared by microwave treatment was 97.4%, and the amount of methanol was only half of the traditional transesterification reaction [74]. The economic analysis of the three methods showed that although the total expenditure of microwave treatment was the most substantial, its net profit was the highest and had significant economic benefits.

### 2.3. Municipal Organic Solid Waste

Municipal organic solid waste refers to the solid waste containing organic substances produced by people in everyday life. The variety of the waste conditions necessitated different treatment methods, which increased the treatment cost. The preparation of organic waste into a catalyst for biodiesel production has been demonstrated to enhance the value of the waste and expanded a new way of disposal. The large-scale use of plastic materials has resulted in significant environmental pollution and has become a global concern. Terephthalic acid was extracted from waste plastics for the synthesis of organic–metal framework (MIL-101 (Cr)) to prepare biodiesel [75]. The B acid strength of plastic-derived MIL-101 (Cr) is slightly lower, and it was observed to coordinate with the L acid formed by unsaturated Cr^3+^ to promote biodiesel production. Despite the observation that both the MOF prepared by plastic-based BDC and MOF crystals made by commercial BDC exhibit a smooth octahedral structure, the crystal size of the MOF prepared by plastic-based BDC is smaller. This has been shown to result in a significant reduction in yield as early as the fourth cycle, due to a reduction in both mass transfer and diffusion. In another study, waste carbon-based catalysts were prepared by calcination and hydrothermal sulfonation of plastics to catalyze palmitic acid distillates [76]. Sulfuric acid sulfonation has been demonstrated to have a significant impact on the morphology of carbon pores and promoted the efficient dispersion of the catalyst pore surface. When the molar ratio of PWC to sulfuric acid was 1:15, the catalyst had the largest pore volume and pore size of 0.071 cm^3^g^−1^ and 6.59 nm, respectively, and the maximum biodiesel yield was 96.9% [77]. However, the optimum temperature for the reaction was 120 °C, necessitating the incorporation of a methanol condensing device or reaction in a closed reactor to prevent the loss of methanol. Armas et al. [78] used Tetra Pak packaging to separate aluminum and carbonaceous materials, subsequently modifying them with KOH or HCl solution to prepare carbon-based catalysts. The TP catalyst was modified with KOH to increase the content of K and O in the catalyst. XPS analysis revealed that K 2p_3/2_ and K 2p_1/2_ were tightly bound to oxygen atoms to reduce the saponification reaction. However, in the transesterification reaction of corozo seed oil, self-toxicity and the poor binding of the catalyst active site resulted in a complete loss of catalytic activity of the catalyst only in the third cycle. Biochar magnetic nanocatalysts were prepared by using cigarette butts as a biochar carrier and SnFe_2_O_4_ as an active material to catalyze the esterification of coconut residue oil. Biochar was doped into SnFe_2_O_4_ to form smaller crystal nanospheres, which enhanced its pore volume. The reaction of reactants with the active sites on the inner and outer surfaces of the catalyst was promoted [79]. The reaction parameters were optimized by RSM-CCD modeling, and the maximum biodiesel yield was 98.67%. Moreover, a high-efficiency catalytic cycle could still be achieved after 7 cycles.

## 3. Inorganic Solid Waste Derived Catalysts

### 3.1. Metallurgical Solid Waste

Various solid wastes generated in the production process of the metallurgical industry are known as metallurgical solid wastes. This mainly refers to the blast furnace slag produced in an ironmaking furnace, steel slag; red mud discharged from alumina refining from bauxite; and various non-ferrous metal slags produced by non-ferrous metal smelting. At present, a large amount of metallurgical solid waste is buried and treated, and it will cause environmental pollution if it is not recycled reasonably. Metallurgical solid waste contains a large number of metal and non-metallic elements, which can provide carriers or active substances for catalysts for the preparation of biodiesel (Table 2).

**Figure 3:**
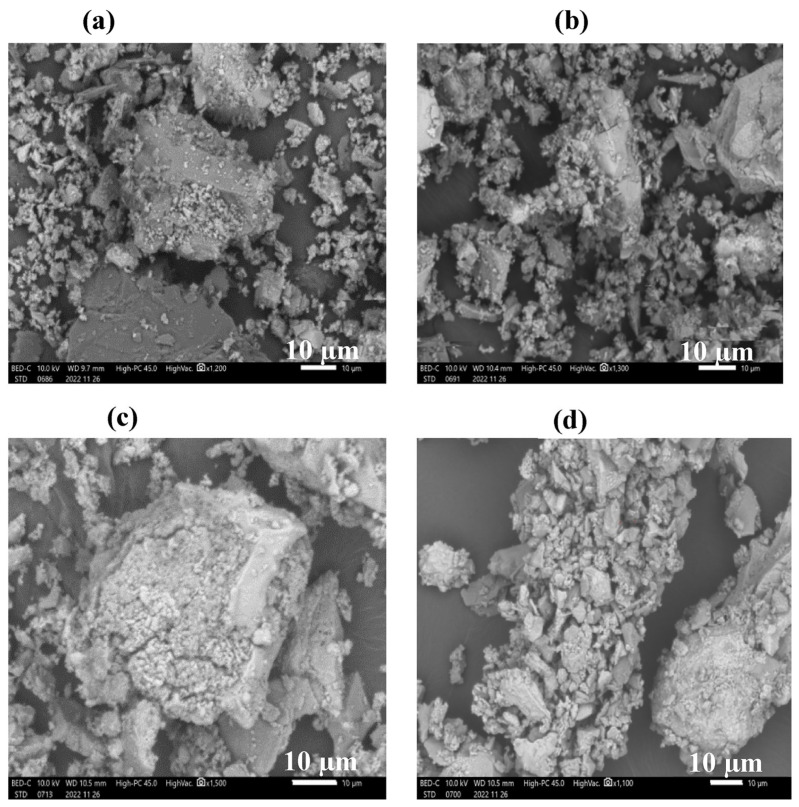
SEM micrographs of BOF-S NC (**a**), BOF-S 850 (**b**), BOF-S 1000 (**c**), and BOF-S 850 after ester exchange reaction cycle 2 (**d**). Reproduced with permission from Reference [91].

The blast furnace slag was calcined directly at 850 °C and 1000 °C to explore the effect of different temperatures on the catalyst [82]. After calcination at a high temperature, the crystallinity of the sample was increased. Silicon ferrite of the calcium and aluminum phase appeared, which led to an increase in the quality of the sample calcined from 650 °C to 950 °C. The SEM images showed that the calcination at 900 °C recondensed the tiny particles and increased the specific surface area (Figure 3b). At 1000 °C, some pores collapsed, although the surface area was slightly reduced, there were more exposed alkaline sites (Figure 3c). Iron oxide provided acidic sites over the catalyst, and the production rate of biodiesel was increased by the synergistic effect with alkaline sites [91]. At the calcination temperature of 850 °C, the maximum biodiesel yield of the catalyst was 90.77%. The overheated modified steel slag was also used to catalyze the pyrolysis of biomass tar, and a loose porous structure was formed under high-temperature calcination, achieving a tar conversion rate of 94.1% [92]. It was confirmed that high-temperature calcination promoted the crystal restructuring of the active material and the modification of the catalyst structure. Iron and steel waste mainly contain C, O, and Fe elements. The alkaline catalyst was prepared by mixing it with calcium nitrate and calcining it [80]. The addition of CaO had highly dispersed flakes on the spherical surface junction after calcination, resulting in a uniform distribution of Fe and Ca elements on the catalyst surface. The effect of Fe on the catalytic ability was much smaller than that of Ca. As the mass fraction of Ca increases from zero, the catalytic ability continues to increase, and the maximum biodiesel yield was 90.48%. The biodiesel yield showed no significant decrease after three cycles. In the recovery and utilization of the catalyst, the washing water led to the denaturation of CaO to form an alkaline environment, which reduced its reusability. If high acid value oil is used as raw material, the carboxylic acid group will adsorb the active site and reduce the biodiesel yield. Therefore, the use of transition metal loading was proposed. After an alkali activation treatment of blast furnace slag, C-A-S-H gel with lower Ca content and higher Al content was produced. If the Al content in the material is too high and contains Mg, a hydrotalcite product will be formed [93]. After the slag was treated with a certain concentration of strong alkali, its crystal structure was transformed into a mixed structure of crystal and amorphous matter showing high alkalinity and high strength. However, the high concentration of strong alkali impregnation increased the solubility of SiO_2_, Ca, and Al. Its liquid had a certain gelation effect, occupying the pores of the catalyst and reducing the pore volume [83]. The predicted biodiesel yield obtained by the CCD fitting quadratic model was 93.15%, and the simulated coefficient of variation was 1.29, proving that the model was reproducible. The Fe element in the electric furnace dust occupied the main part, and it was mixed with reed straw to prepare a magnetic biochar-based catalyst [89]. The Fe element was calcined to synthesize a magnetic material to provide stable magnetism for the catalyst. By comparing the old and new catalysts by ICP, it was found that the percentage of sodium decreased. Therefore, it can be inferred that the active sites in the catalyst were mainly provided by sodium. In high-temperature calcination, reed straw as the main carbon source of the catalyst and the impregnating agent NaOH formed the Na_2_CO_3_ active substance to promote the transesterification reaction. The catalyst had an extremely high biodiesel yield (99.89%) and cycle times (93.61% after 11 cycles). They were higher than the yield and cycle times of biodiesel catalyzed by KNO_3_/BC-Fe_2_O_3_ [94], Fe_2_O_3_/Fe_2_K_6_O_5_ [95], Na_2_SiO_3_@Ni/C [96], and other catalysts, making it cost-effective.

**Figure 4:**
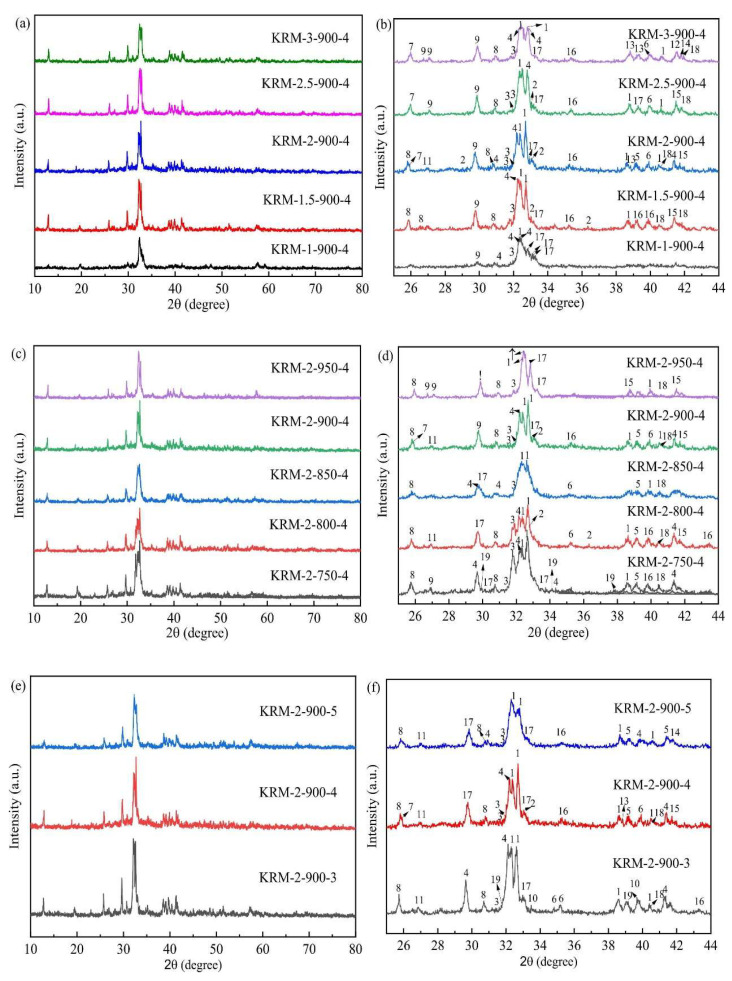
XRD patterns of red mud-based potassium composite catalysts: (**a**,**b**) different K_2_CO_3_/RM mass ratios (1:1, 1.5:1, 2:1, 2.5:1, and 3:1), (**c**,**d**) different calcination temperatures (750, 800, 850, 900, and 950 °C), (**e**,**f**) different calcination times (3, 4, and 5 h). Reproduced with permission from Reference [85].

Red mud (RM) is mainly composed of fine particles containing aluminum, iron, silicon, titanium oxides, and hydroxides. The dry red mud was crushed and calcined to study the effect of varying temperatures on the performance of the catalyst. The biodiesel yield of more than 94% was achieved at 200 °C calcination. As the calcination temperature increased, the mineral phase in the catalyst was destroyed, and no active substance was detected [84]. Due to the high alkali leaching ability of the catalyst, the catalytic activity was reduced, which made the catalyst unrecyclable. The catalyst could only be involved in the reaction by impregnating the active material again. This increased the number of preparation steps and was not conducive to the promotion of commercialization. Environmentally friendly precursors were introduced to reduce the cumbersome and polluting effects of solid acid catalyst preparation and improve its catalytic performance [97]. The biomass (acai seed) and red mud were mixed in different proportions, calcined, and then activated by sulfuric acid to prepare carbonaceous magnetic acid catalyst [81]. Different proportions of magnetic source (Fe_3_O_4_) and sulfonic acid groups determined the active sites on the catalyst. The higher calcination temperature removes the attachment and decomposition of sulfonic acid groups, so it was suitable for the reaction temperature of 150 °C. The conversion rate of oleic acid to methyl oleate was 88%, and it can be recycled three times. In another study, the catalysts were prepared using the solid phase method by thoroughly mixing dried red mud with K_2_CO_3_ and calcining [87]. In the XRD pattern, it was found that K salt and Ca salt together constitute the active component, and reached the maximum peak strength at 900 °C and 4 h calcination (Figure 4). Through thermodynamic calculation, ΔH = 47.02 kJ/mol, and ΔG = 82.85 kJ/mol. The low comparison with other catalyzed castor oils suggests that this catalyst can perform transesterification reactions more easily. The same method was used to replace K_2_CO_3_ with Li_2_CO_3_ to prepare the catalyst. Due to the small radius of lithium ions, it was easy to enter the framework of Al_2_O_3_ and Fe_2_O_3_ to form O^2−^. O^2−^ can also provide adsorption sites for methanol to promote methanol reaction. Although ΔH = 54.04 kJ/mol and ΔG = 93 kJ/mol were greater than the red mud catalyst mixed with K_2_CO_3_, the maximum biodiesel yield was 96.32%, and it was reused four times. Ghazala et al. [86] used the sol–gel method to prepare Al_2_O_3_ from waste aluminum and then loaded CaO to prepare an alkaline nanocatalyst. The catalyst had a stable alkalinity measured by the reaction titration method. Al_2_O_3_ as a carrier made the Ca ions in CaO have a lower binding energy, which can improve the alkalinity of the catalyst. The catalyst was regenerated after each cycle, and the biodiesel yield after five cycles was higher than 90%. The CaO/Al_2_O_3_ catalyst prepared by the impregnation method can achieve a biodiesel yield of 94%, but its reaction time of up to 5 h consumes more energy [98].

### 3.2. Coal-Fired Solid Waste

Coal-fired solid waste refers to a large amount of solid waste such as fly ash, slag, and desulfurized gypsum produced by coal-fired power generation enterprises in the production process. Among them, fly ash gold and slag contain a large amount of metal oxides (SiO_2_, Al_2_O_3_, CaO, MgO, etc.). Using these solid wastes as catalysts for biodiesel can not only reduce costs but also reduce environmental pollution.

Fly ash is composed of SiO_2_ and Al_2_O_3_, which has high thermal stability and can be used as the carrier material of the catalyst. The performance of the catalyst can be improved by adding some active metal ions by the impregnation method. By impregnating fly ash with Zn(NO_3_)_2_ by calcination at 400 °C, it was found that the hexagonal wurtzite structure of ZnO and ZnFe_2_O_4_ made the catalyst have a more complex pore size distribution [99]. Compared with the sponge-like structure of fly ash-loaded CaO with aggregates, its surface area increased by 8 times [100]. However, the yield of biodiesel decreased rapidly in the reusability experiment. In the later stage, the use cycle of the catalyst can be improved by optimizing the ratio of ZnO to the carrier. Doping metal ions can not only change its structure, but some metal ions can also increase the alkalinity of the catalyst. Loading LiNO_3_ onto fly ash can increase its alkalinity. Where calcined LiNO_3_ is decomposed, O Lewis alkali anions are associated with the production of moderately alkaline sites, which can improve the yield of glycerol carbonate [101]. However, loading that is too high leads to delayed surface desorption of strong ions, so the optimum loading is chosen to be 2 wt%. The Li-O-Si bond formed by the catalyst activates the hydroxyl group of the waste glycerol, and the carbonyl carbon of the dimethyl carbonate reacts with the glyoxylate to form glycerol carbonate. A maximum conversion rate of 96% is obtained under the optimal conditions. The yield of biodiesel is higher than that of the Li-doped ZnO catalyst, and the high temperature calcination makes Li replace Zn as a strong alkaline site, which confirms the strong catalytic activity of Li [102]. The catalyst was prepared by hydrothermal treatment of fly ash under alkaline conditions and then activation with potassium nitrate [103]. Under high temperature calcination, the alumina decomposed by mullite reacts with potassium nitrate to form KAlO_2_, a strong catalytically active material to promote the transesterification reaction. Under ultrasonic treatment, the catalytic reaction is completed in only 1.4 min, and the yield of biodiesel can still be maintained at more than 90% after 8 times of repeated use, which has a good application prospect.

Zeolite is a microporous crystalline aluminosilicate, which is mainly synthesized by the precipitation of silicon and aluminum. Fly ash contains a large amount of silicon dioxide which can be used as the silicon source of zeolite [104]. Zeolite is prepared by mixing silicate solution and aluminate solution to form gel. The maximum biodiesel yield obtained from transesterification of soybean oil using a 4 wt% loading catalyst at a reaction time of 2 h was 95.5%. NaOH was impregnated on the surface of zeolite, which effectively reduced the number of strong acid sites to form a mesoporous structure. Hydrothermal treatment promotes rapid nucleation in fly ash to form a rough surface. The Si-O-Na group formed by it acts as an active site during the transesterification process and was used to produce active substances [105]. The zeolite-based catalyst prepared by the hydration-dehydration method combines eggshell and fly ash. Its high alkalinity promotes the conversion of sunflower seed oil to biodiesel. An alkali solution promotes the conversion of aluminosilicate into zeolite. High temperature calcination makes the structure of zeolite transition to a rod-like particle structure and no agglomeration occurs, which is conducive to the adhesion of active substances [106]. After the reaction, the catalyst can be separated and used continuously five times, and its FAME content is still 97.9%. The two-phase CaO/Ca_2_SiO_4_ mixture in the calcium silicate compound synthesized by solid phase reaction has catalyst activity, and the conversion rate of triglyceride can reach more than 96% [107].

### 3.3. Chemical Solid Waste

Chemical solid waste refers to the solid waste in the chemical industry production, which cannot be reused, needs to be recycled, treated or sterilized. It mainly includes waste chemicals, expired drugs, polluted wastes, and so on. Chemical solid waste is characterized by diversity, danger, and difficult degradation. The added value of pharmaceutical waste can be increased by producing catalysts from them.

Previous studies have found that the performance of biodiesel prepared with MgO as a catalyst was better than that of biodiesel prepared with CaCO_3_ as a catalyst by GC-MS [108]. The catalyst was synthesized using waste magnesium oxide and calcium carbonate tablets as raw materials, which were employed to catalyze the conversion of waste oil into biodiesel. The calcined catalyst had a layered structure, showing smaller and denser particles. Its specific surface area of 12.72 m^2^g^−1^ was higher than that of separately calcined magnesium oxide tablets and calcium carbonate tablets, which promotes the adsorption of active substances. The highest percentage of biodiesel yield obtained under the best conditions was 96%, which was reduced to 71.4% after four cycles and had good thermal stability [109]. Nevertheless, in comparison with the ASTM standard, concentrations of H and O below the standard may cause incomplete combustion and pollute the environment. Zhang et al. [110] investigated the effect of catalysts with different ammonium molybdate loadings using urea as carrier on the catalytic ability of waste soybean oil. Mo^6+^ oxides, as catalytically active substances, exhibited high crystallinity and existed in catalysts with high Mo loading. However, XRD results showed that the metal dispersion decreases when the Mo loading is greater than 3%. In particular, the SEM pattern showed small particle agglomerates on the surface of the catalysts with 10% loading. Heavy metal pollution and low biodiesel yield of molybdenum-based catalysts are the main reasons their development is limited. The biodiesel yield of the catalyst was 84.8%, which was higher than 82.1% of BMK10 [111] and 79.7% of Sr/ZrO_2_, showing a good development prospect. In another study, Mekonnen et al. [112] used different methods (untreated, thermally activated, and NaOH-impregnated thermally activated) to treat Ca-containing precipitates in bleaching agents. In the SEM image, it was found that only thermal activation would cause agglomeration. And after NaOH impregnation and thermal activation, it showed a mist-like arrangement of blocks, providing sites for more active substances to adhere. The impregnation of alkaline solution complements the shortcomings of low catalytic activity of CaO, and also contributes to the formation of the core structure of the calcined catalyst. The yield of biodiesel catalyzed by CaO nanocatalyst was 67% [113], and the biodiesel yield of the catalyst can reach 76.05%, which has certain feasibility. However, the lower cetane number needs to be paid attention to in later research.

### 3.4. Construction Solid Waste

Dirt, waste concrete, waste masonry and other wastes generated by people engaged in demolition, construction, renovation, repair, and other production activities in the construction industry are collectively referred to as construction solid waste. The main mineral phases are quartz, calcite, sodium/calcium aluminosilicate, albite and portlandite, etc. [114]. The main elements are calcium, silicon, aluminum, etc., which can be used as precursors to prepare active catalysts.

**Figure 5:**
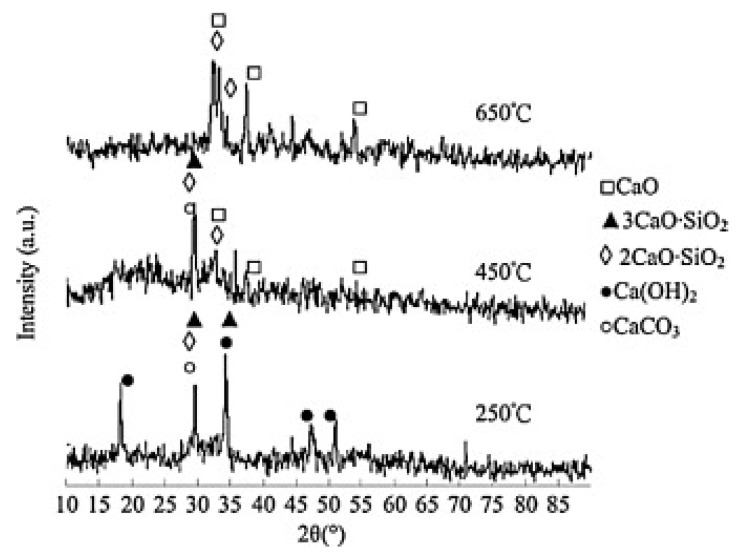
XRD patterns of hydrated cement obtained by calcination within the range of 250–650 °C. Reprinted with permission from Reference [116].

C & D waste (i.e., concrete and mortar) collected on construction sites was activated at 850 °C calcination temperature [115]. Due to the existence of aggregates in mortar and concrete catalysts, the porosity was low, and the final transesterification efficiency was only 39 ± 0.2% and 30 ± 0.3%. Wang et al. [116] calcined cement at different temperatures as a catalyst for catalyzing soybean oil. The results showed that CaO was produced after a calcination temperature above 450 °C (Figure 5). Its alkalinity ranges from 15 to 18.4, which leads to the high catalytic activity of the catalyst. The higher calcination temperature weakens the bond strength of the concrete and makes the crystal plane break. Therefore, the best conversion rate of fatty acid methyl ester can reach 97.6% by calcining the catalyst at 650 °C. The CaO catalyst prepared by the simple calcination of limestone reached a biodiesel yield of 90% [117], and it was verified that the catalyst CaO could be combined with glycerol to prevent oxidation by air, which was also the advantage of the catalyst with CaO as the active material in actual production.

A catalyst consisting of a mixture of other wastes with construction solid waste promoted a complementary structure and produced a number of positive effects. An acid-base bifunctional catalyst was prepared by direct hydrothermal sulfonation of coffee grounds and cement slag. Sulfonation of sulfuric acid was beneficial to the formation of oxygen groups and amorphous carbon structure of the catalyst [118]. It also enhanced the adhesion of sulfonic acid groups on the surface of the catalyst. After four catalyst cycles under the optimal conditions, the EDX mapping showed that the active substances containing S, Ca, and Si were leached. This resulted in a reduction in biodiesel conversion from 92.3% to 79.4%. The CaO/Ag nanocatalyst prepared by Zhu et al. [119] achieved a biodiesel yield of 90.95% in the first catalytic process and decreased to 76.69% after 5 cycles. The metal salt can change the acidity and alkalinity of the catalyst prepared by mixing marble and horn [120]. The SEM images also showed that after calcination, the catalyst exhibited irregularity and the pore size increased, which was mainly because the calcination failed to completely remove the residual carbonate. After the artificial neural network simulation of the biodiesel production process and the optimization of reaction conditions, the maximum biodiesel yield was 98.13% and could be recycled 7 times, which demonstrated the strong stability of the catalyst.

### 3.5. Mining Solid Waste

Tailings and waste rock produced during the mining and washing of ores are known as mining solid waste. Tailings are usually composed of small rock materials and waste water produced by processing plants. They are characterized by both organic and inorganic compounds.

Prates et al. [121] compared three different preparation methods to prepare sulfated iron oxide catalysts using iron ore waste as a raw material. The treatment of ammonium sulfate and sulfuric acid did not change the structure of the iron ore waste, but only increased the acidic sites of the catalyst. The esterification reaction of the three catalysts was carried out. It was found that the sulfuric acid-treated catalyst could not only achieve a 100% conversion rate but also had higher kinetic parameters and the shortest reaction time. Sulfuric acid dissolved Fe^3+^ in hematite waste ore and formed alkaline acid sites after calcination at a high temperature. However, the low acidity of ammonium sulfate was not effective at leaching waste ore to form an active phase. The sulfate group was easily leached by methanol, resulting in a significant decrease in its second cycle efficiency and no good reusability.

Dolomite is a natural source of calcium. The catalysts were prepared by co-precipitation or a hydrothermal synthesis method by mixing with dolomite using sapindus solution as the activating substance [122]. The sapindus solution could effectively reduce the particle size of the catalyst and prevent particle agglomeration, forming a cubic structure. Hydrothermal treatment promoted the regular growth of catalyst crystals, and the particle size was only 1 to 2 μm [123]. The CaO-MgO catalyst prepared by directly calcining limestone had a biodiesel yield of up to 75%, while the catalyst could achieve a biodiesel yield of 92.40%. Cerium was incorporated into dolomite by direct wet impregnation, solid mixing and wet impregnation, respectively. The catalyst prepared by wet impregnation had the highest biodiesel yield and the largest basic density. Oxygen vacancies were created due to more Ce^3+^, leading to the appearance of broken holes [122]. Cerium nitrate with calcium and magnesium oxides appeared as an irregular lamellar structure under high temperature calcination. This enhanced the catalytic ability of the catalyst. The maximum biodiesel yield of 97.21% was higher than the maximum biodiesel yield of 90.1% obtained by using the modified TiO catalyst [124]. Because the interaction between CaO and CeO also reduced the leaching of more active substances, the biodiesel yield was 88.63% after five cycles.

**Figure 6:**
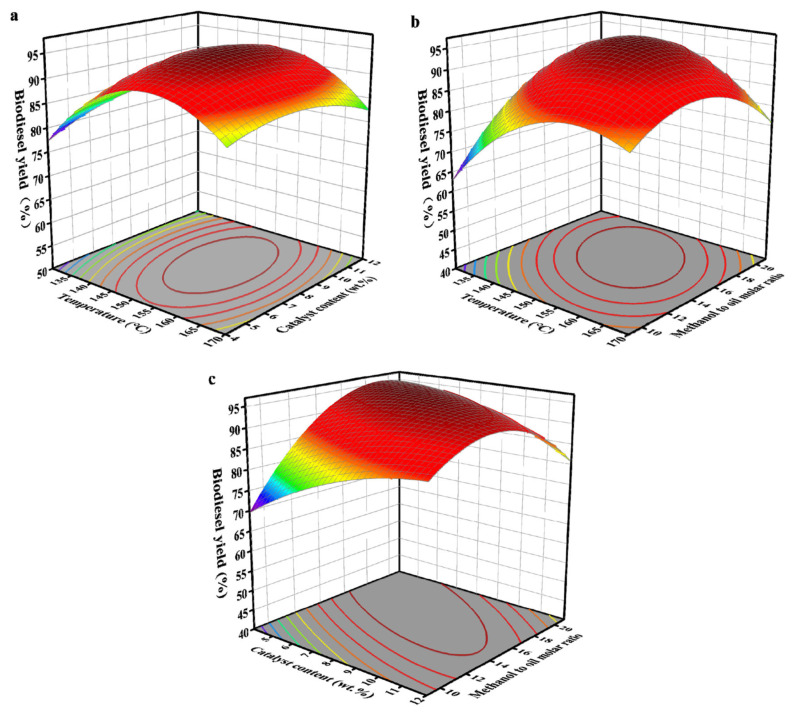
Surface view of the interaction between exchange conditions and catalytic performance. (**a**) Temperature and catalyst content; (**b**) Molar ratio of methanol to oil and temperature; (**c**) Molar ratio of methanol to oil and catalyst content. Reprinted with permission from Reference [126].

Wollastonite is a kind of calcium silicate mineral, which is usually flaky, radial or fibrous. It has the characteristics of non-toxicity, chemical resistance, and thermal stability [125]. Metal ions can change the acidity and alkalinity of the catalyst. Sr doping helped to improve the alkalinity of the catalyst. However, a high doping amount causes a saponification reaction. La doping can reduce the alkalinity of the catalyst and interfere with its activity. Nevertheless, the addition of 5 w% oleic acid to palm oil resulted in an enhancement of the acid resistance of the catalyst [126]. The biodiesel yield was found to be capable of achieving 91.2%, thus demonstrating the efficacy of this process. RSM modeling was used to optimize the process parameters of the transesterification process (Figure 6). When the catalyst loading was 6.4 wt%, the reaction temperature was 149 °C, the molar ratio of methanol to oil was 17:7, and the predicted biodiesel yield was 96.13%. Although the prepared L-B bifunctional acid nanoclay catalyst had a disordered layered structure, its maximum biodiesel yield of 93.1% was still lower than that of the Sr-La bifunctional catalyst [127]. In another study, CaO was loaded into wollastonite as an active material to explore the effect of CaO loading on the catalytic effect [128]. When the mass ratio of CaO to wollastonite was 0.8, SEM images revealed that a transformation in the catalyst structure changed from needle-like or plate-like to rod-like. An optimal amount of Ca enables uniform dispersion on the silicon surface, reducing particle agglomeration. However, an excessively high Ca-to-wollastonite mass ratio leads to active sites being encapsulated by calcium, forming block-like particles and reducing the catalyst’s alkalinity. The maximum biodiesel yield of the final catalyst reached 97.59%, and after five cycles, a biodiesel yield of 87.30% was still achieved, demonstrating the catalyst’s stability and feasibility.

### 3.6. Restaurant Solid Waste

#### 3.6.1. Eggshells

The egg shell of oviparous animals is called eggshell, which is non-toxic, easily manipulable, inexpensive, and renewable. The main component of the eggshell is CaCO_3_. The substance can be subjected to calcination at an elevated temperature in order to yield calcium oxide (CaO). It is a heterogeneous Ca-based catalyst commonly used in biodiesel production (Table 3).

Tshizanga et al. [131] found that eggshells calcined at 800 °C exhibit microcrystalline structures, which increase the surface area of the catalyst and enhance the attachment of active sites. The maximum biodiesel yield achieved was 91%, which is lower than that of the catalyst developed by Sai et al. [142] using eggshells calcined at 900 °C (97.84%). However, the catalyst demonstrated excellent stability, capable of being continuously recycled for 10 cycles. Amal and other studies have shown that the calcination of the eggshell and the doping of transition metals (Fe, Ni) can improve the strength of the catalyst [129]. SEM confirmed that the structure of the catalyst was dense, and the surface was porous. BET calculated that the catalyst has a surface area of 9.19 m^2^/g and a pore volume of 0.0022 cm^3^/g, which promotes the reaction of triglyceride (TG) molecules with methanol. On the other hand, catalysts were prepared by doping Na/K in CaO [135]. The surface area was 12.6 m^2^/g and the pore volume was 0.03 cm^3^/g as detected by BET-BJH, which was larger than that of the catalyst doped with Fe. Its highest yield reached 97.6%, which had good application prospects. In addition to doping metal ions, the catalyst prepared by eggshell can also be doped with metal oxides. This not only increases the acidic sites of the catalyst but also reduces the pollutant emissions of biodiesel. The magnetic Ca-based catalyst was obtained by doping the metal oxide MnFe into the calcined eggshell and chicken bone powder [141]. The incorporation of MnFe and K metal oxide particles results in larger particles and fewer pores, but the metal elements in the catalyst can enhance the electrophilicity of the active sites and promote the attack of methanol on carbonyl carbon. The GC mass spectrometry analysis showed that 99% of the fatty acids in the waste edible oil were converted into methyl esters. The flash point of biodiesel was 162 °C, reaching three times that of diesel.

**Figure 7:**
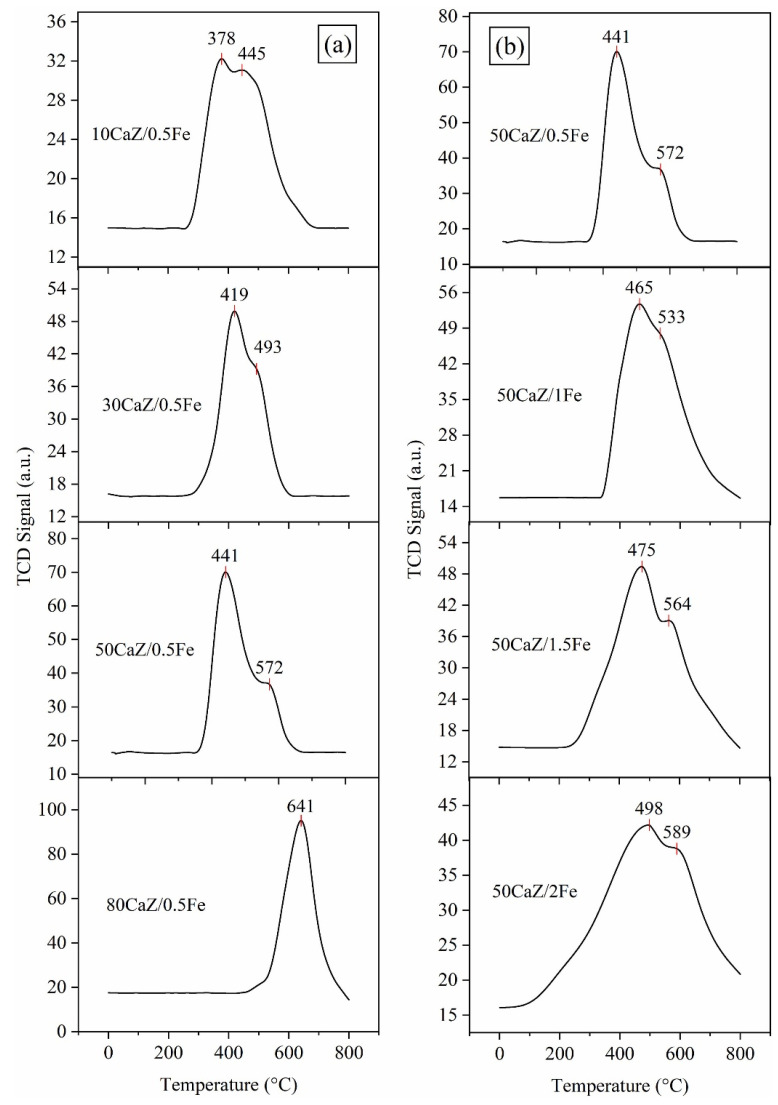
TPD-CO_2_ spectra of the catalyst under different (**a**) CaO and (**b**) Fe_3_O_4_ loadings. Reproduced with permission from Reference [132].

Mohammed A.S. et al. [138] produced CaO nanocatalysts by hydration-dehydration technology. From the SEM results, it was shown that the catalyst surface had a particle shape. Inside, a large number of cavities were created by the escape of gaseous water molecules due to the decomposition of Ca(HCO_3_)_2_. This promotes the contact between the reaction sites and the reactants. Under the optimal reaction conditions, the maximum yield of croton biodiesel was 98.314%. The catalyst could be reused six times and showed good stability. In another study, the catalyst was prepared by using activated zeolite and ground eggshell [134]. One of the 2 g zeolite-loaded catalysts exhibited a high surface area of 83.6 m^2^/g and a high alkalinity of 1.07 mmol/g. Due to the interaction between CaO and zeolite at 800 °C calcination, CaO is adsorbed on the surface of zeolite. Through the analysis of the TPD diagram, the wide desorption peaks corresponding to 300 °C and 566 °C confirmed the existence of the strongest basic sites in the 2ZE/ES-800 catalyst. Lani et al. [132] prepared magnetic zeolite-loaded catalysts by the hydrothermal method for use in the transesterification reaction of waste cooking oil. This included the synthesis of CaO calcined from eggshells and ZSM-5 molecular sieves produced from rice husk and mixed with Fe_3_O_4_. The loading of CaO and Fe_3_O_4_ not only increased the number of active sites but also its own rough surface increased the surface area of the catalyst. However, excess CaO or Fe_3_O_4_ densified the catalyst and reduced the total surface area of the active site blockage (Figure 7). The biodiesel yield (91%) and recovery (88%) of the 50CaZ/0.5Fe catalyst were much higher than those of the CaO catalyst, confirming its remarkable stability.

In order to reduce the high energy consumption of biodiesel prepared by traditional methods, ultrasonic-assisted and microwave-assisted methods were widely used in the production of biodiesel. Chen et al. [143] explored the use of calcined ostrich eggshell to obtain CaO for the ultrasound-assisted transesterification of palm oil. The energy of ultrasound can emulsify the reactants to give them a large surface area. It also facilitated the attachment of active substances and increased the rate of the transesterification reaction. A biodiesel yield of 92.7% could be obtained using only 60% ultrasonic power. Microwave-assisted energy transfer at the molecular level enabled efficient heating and greatly shortened the reaction time. Zhang et al. [144] used the microwave-assisted conversion of chicken feather meal oil into biodiesel. Microwave heating can reduce heat loss; however, excessive power can raise the reaction temperature above the optimal level, making it easier for glycerol to hydrolyze into free fatty acids. The maximum biodiesel yield of 95% can be obtained in only 5 min at 500 W power.

#### 3.6.2. Shellfish

Shellfish is a kind of mollusk, usually wrapped in the shell of mollusks. There are more than 110,000 species of shellfish. The waste shell contains about 95% CaCO_3_, which can be applied to the preparation of Ca-based catalysts. The crab shell was calcined at 900 °C to prepare heterogeneous solid catalyst for esterification reaction [145]. Calcination activated the porous structure of the catalyst, so that the pore volume of the CaO catalyst was 0.032475 cm^3^/g, and the contact area with karanja oil was increased. The molar ratio of methanol to oil was 8:1, and the reaction time was 120 min; catalyst loading was 2.5 wt%; under the reaction temperature of 65 °C and stirring rate of 700 rpm, the yield of biodiesel was 94%. Singh et al. [146] studied the effect of *Angulyagra oxytropis* and *Bellamya crassa* snail shells on the performance of the catalyst at different calcination temperatures. In the XRD pattern, it was confirmed that at 900 °C, the Ca nucleation grain size of the two snail shells was smaller. During the calcination process, the release of CO_2_ and the crystal growth of CaO increased the porosity of the catalyst. However, the high temperature calcination at 1000 °C was easy to produce a sintering effect, which reduced the surface area of the catalyst. Two kinds of snail shells can achieve optimal methyl ester harvesting rates of 95.87% and 95.91% It could be recycled up to 5 times and was also somewhat stable. In the transition metal doped calcium-based catalyst, the reaction activity was low due to the low alkalinity of the transition metal. Jindapon et al. [147] used a dissolution-precipitation method, using shells as raw materials doped with Zn and Al compounds to prepare catalysts. The XRD confirmed that the interaction between CaO and Al_2_O_3_ was enhanced, which reduced the diffusion of CO_2_ and H_2_O in the catalyst and maintained the stability of the catalyst in air. Among them, the ZSA-500 catalyst showed a maximum biodiesel yield of 98.0 ± 1.60 wt% and could be reused 5 times. Biodiesel analysis showed that the contents of monoglyceride, diglyceride, and triglyceride were 0.02 wt%, 0.06 wt%, and 0.01 wt%, respectively. All are below the standards set by the Thai Ministry of Energy. In another study, oyster shells were used as catalysts to produce biodiesel in a microwave heating system. The green production of biodiesel was achieved by reusing oyster shells [148]. At different reaction temperatures, the regression equation under the kinetic model was determined as a line and the regression coefficient R^2^ = 0.9994. According to the Arrhenius plot, the activation energy Ea = 9.58 kJ/mol. The smaller the activation energy, the easier the reaction. Under the optimal reaction conditions, the maximum biodiesel yield was 91.1%, which was similar to 90.3% of the AC-600-SO_3_H@Fe/C catalyst [149]. However, it lost activity in only 3 cycles, which was speculated to be related to the leaching of active substances.

#### 3.6.3. Animal Bone

**Figure 8:**
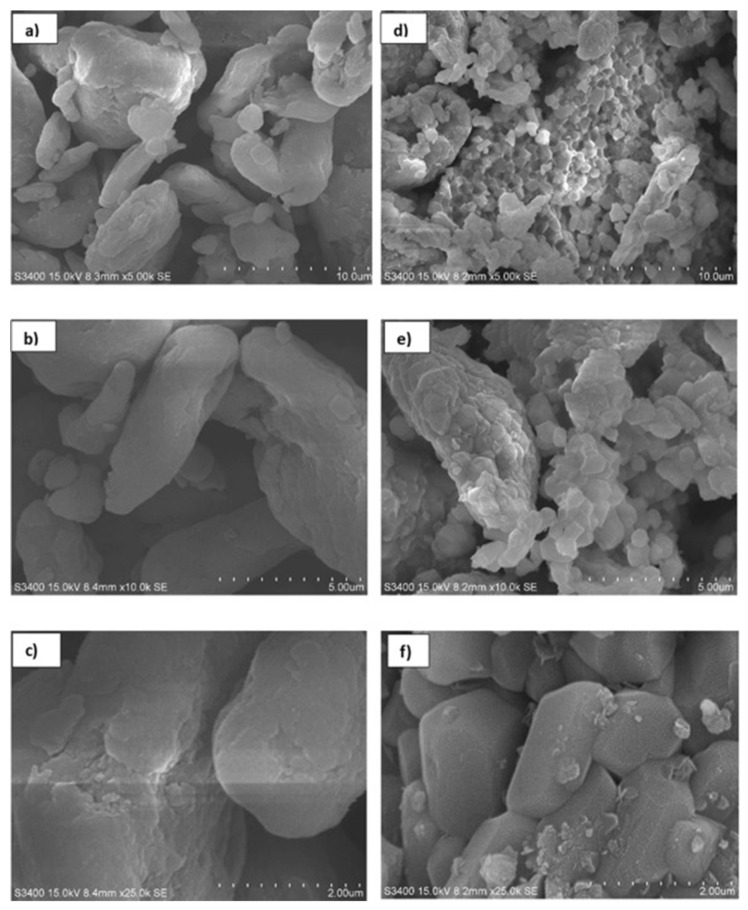
SEM images of (**a**) 10 μm, (**b**) 5 μm, and (**c**) 2 μm uncalcined samples, and (**d**) 10 μm, (**e**) 5 μm, and (**f**) 2 μm calcined samples (900 °C). Reproduced with permission from Reference [150].

Animal bones are hard tissues in animals, which have the function of supporting the body, mainly with CaCO_3_ as the main component. Animal bones are a kind of solid waste produced by meat. Because they contain hydroxyapatite, they can be used to prepare catalysts to produce biodiesel. This not only reduces waste disposal but also allows for green energy production (Table 4). Khan et al. [150] used the catalyst derived from waste ostrich bones to prepare biodiesel in waste edible oil. The effect of different calcination temperatures on the catalytic performance was studied. It was found that the catalytic performance increased with the increase in calcination temperature. The study demonstrated enhanced catalytic activity at 900 °C, yielding a biodiesel yield of 90.56%. The calcination at higher temperatures reduces the specific surface area of HAp particles, which was mainly due to the sintering phenomenon of the catalyst. The specific surface area of the catalyst calcined at 900 °C was 3.61 m^2^/g. Although the specific surface area was small, the alkalinity was improved. The morphology of calcined and uncalcined catalysts was analyzed by SEM. The uncalcined catalyst showed an amorphous crystal structure. while the calcined catalyst showed a hexagonal crystal structure with a reduced particle size and regular shape (Figure 8d–f). The activity of the catalyst was improved by mixing a variety of wastes. Chicken claw bones, catfish bones, and two mixtures were calcined at a high temperature of 1000 °C to develop Ca catalysts [151]. Comparing the FTIR of the three catalysts, it was found that only the CMCP showed the presence of amines and amides. It proved that the solid–solid adhesion was increased after mixing. Moreover, the SEM images showed that the CMCP had less fractures and a uniform shape, which was also the reason for the presence of covalent bonds in the CMCP. The CMCP exhibited excellent catalytic performance, and the biodiesel yield decreased from 94.7% to 90.1% after 5 cycles.

The catalyst was activated by various reagents, and potassium salt had high catalytic activity. Erfan Mohebolkhames et al. [152] prepared alkaline catalysts by calcining salmon bones, impregnating them with KOH solutions of different concentrations, and then calcining them again. FESEM revealed that the KOH (40–873)/SFB3 catalyst exhibited an aggregated morphology. Approximately 60% of the particle sizes were in the range of 20–40 nm, indicating that this catalyst was a compact nanomaterial. Under the optimal conditions, the catalytic yield of biodiesel reached 99.13 ± 0.8%. Due to the leaching of potassium and other oil or the heterogeneous blocking of active sites, the catalyst could be reused four times and the yield decreased to 89.76%. Maleki H. et al. [153] studied CaO-La catalysts impregnated with Li. The doping of Li greatly increased the alkalinity of the catalyst, achieving a biodiesel yield of 93.5% in the first cycle. Esther O. Babatunde et al. [154] studied a new method: extracting SiO_2_ from a termite mountain and doping it into bovine bone to prepare the catalyst. SiO_2_ had a sufficient surface area to adhere to the metal. The BET analysis results showed that the calcined catalyst has a high surface area of 440.88 m^2^/g. Through the kinetic and thermodynamic analysis of the transesterification process, the activation energy Ea of the reaction was calculated to be 41.4 kJ/mol. The lower activation energy promoted the rapid reaction. Under the optimal conditions, the conversion rate of biodiesel was up to 95.12%, and it could be stably cycled for 3 times. In another study, Mariam AlSharifi et al. [155] prepared different amounts of lithium nitrate loaded on chicken bones by the impregnation method. At a lithium loading of 2 g and a calcination temperature of 850 °C, thermal gravimetric and X-ray diffraction analyses revealed enhanced crystallization of CaO in the chicken bone catalyst. Rod-shaped particles were formed in the microstructural images. The doping of Li^+^ increased the crystallinity of the catalyst, but also inhibited the intensity of the CaO peak. The catalyst was used to catalyze waste rapeseed oil and fresh rapeseed oil. Under the optimal conditions, the FAME content was 94.9% and 96.6%, respectively. This confirms that even under conditions of high acidity, the catalyst is still able to perform its catalytic function. Rajat Chakraborty et al. [156] developed catalysts for loading different antimony chlorides into pig bones. An infrared radiation (IRI) auxiliary reactor was added to the esterification reaction, which reduced the reaction time. IRI penetrated energy into the reaction mixture, resulting in strong molecular collisions. Compared with traditional heating, the conversion rate of IRI increased by 13%. At the end of each cycle, the catalyst was separated, washed, and dried for the next experiment. The catalyst could be reused up to eight times and the conversion rate was maintained at 99 ± 1%, showing strong stability.

**Table 4:** Production of biodiesel using various animal bone-derived solid alkali catalysts and different raw materials via transesterification.

No.	Catalyst Source	Feedstock	Catalyst	Optimum Conditions				Yield (%)	Citation
				A	B	C	D		
1	Beef bone	*Palm fatty acid distillate*	CaSO_4_	20:1	5	180	70	81.5	[157]
2	Goat bone	*Goat fat oil*	CaO	12:1	2.5	120	60	87.2	[158]
3	Camel bone	*jujube seed oil*	HAP	7:1	4	180	75	89	[159]
4	Chicken bone	UCO	CaO	15:1	5	240	65	89.33	[160]
5	Animal bones and teeth	*Castor oil*	CaO/P_2_O_5_	9:1	5	180	60	89.5	[161]
6	Ostrich bone	UCO	HAP	15:1	5	240	60	90.52	[150]
7	Ox bone—Termite Mountain	UCO	CaO/SiO_2_	9:1	2	150	65	95.12	[154]
8	Animal bones	*Neem oil*	CaO/K	9:1	6	180	70	96.01	[162]
9	Chicken bone	*Rapeseed oil*	CaO/Li-Cb	18:1	4	35	60	96.6	[155]
10	Animal bones	*Neem oil*	CaO/K	12:1	5	240	65	96.82	[163]
11	Chicken bones—catfish bones	*Mahogany oil*, *Brazilian rubber tree oil*, and *Guinea rubber tree oil*	CaO	4.62:1	4.16	69.76	69.79	97.12	[151]
12	Salmon bones	*Sunflower oil*	CaO/K	10:1	10	180	65	99.13	[152]

A: molar ratio (methanol:oil); B: catalyst loading (wt%); C: time (min); D: temperature (°C).

## 4. Prospects for Solid Waste Applications

### 4.1. Cost Analysis

The cost analysis of using solid waste as a catalyst to produce biodiesel involves the estimation of asset investment, operation and maintenance, and revenue. It can determine the feasibility of large-scale commercial application. It has been determined that the raw material price accounts for about 70% of the production cost of biodiesel and biodiesel production technology [164]. This is the most significant impediment to the commercialization of biodiesel. In order to highlight the economy of biodiesel, it was pointed out that the use of low-cost materials such as eggshells, animal bones, biochar from diverse plant sources and various industrial waste products can be used as raw materials for catalyst preparation. That is, the production cost of biodiesel can be minimized through the utilization of heterogeneous catalysts derived from organic or inorganic waste. Zhu et al. [119] prepared a CaO/Ag catalyst to produce soybean biodiesel. By calculating the cost of raw materials, transportation, labor, and capital investment, we found that each kilogram of biodiesel requires USD 1.68. This was found to be less than the price of diesel produced from petroleum. The CaO catalyst produces soybean biodiesel at only USD 1.29 per kilogram. Although the biodiesel produced using the CaO/Ag catalyst is more expensive, the CaO/Ag catalyst produces fewer raw materials and is more efficient. The high cost of the catalyst is mainly due to the incorporation of Ag precious metals, and subsequent replacement of these metals with less expensive alternatives has been identified as a means of reducing costs. In order to improve its economic feasibility, it is necessary to consider the source of raw materials, the preparation process of the catalyst, and the number of reactants in the production process. The necessity for this research was predicated on the development and requirement of Nigeria. Oke E.O. et al. [165] used ABPD to carry out scale-up simulation and economic evaluation of biodiesel production from neem oil. After setting the production requirements of 3300 tons of biodiesel, the ASPEN algorithm gave 251 batches/year, 13,147 kg/batch, and the production cost of biodiesel was determined to be USD 1.06 per metric ton. The annual income of biodiesel production is USD 6,113,750, and the annual income of its by-product (glycerol) is USD 418,750. The production arrangement is expected to be used for 30 years and the payback period is 2.67 years. The feasibility of producing biodiesel from neem oil was ascertained through a comprehensive analysis of the profitability sensitivity of the model. When the sales price of biodiesel increases from USD 1.5/kg to USD 1.9/kg, the return on investment increases from 30.3% to 48.3%. When the sales price is less than USD 1.5/kg, the NPV is negative. When the total production cost increases from USD 2.12 million to USD 4.95 million, the payback period increases from 1.66 to 7.31. When the total production cost exceeds USD 4.95 million, the NPV is negative. Minimizing the total production costs frequently results in enhanced profitability [165]. The equipment cost accounts for the largest proportion in the whole process of biodiesel production, and the ultrasonic treatment system is cheaper than the traditional heating and stirring system. The use of ultrasound-assisted biodiesel has been demonstrated to facilitate the amalgamation of reactants and accelerate production efficiency. The production process was inefficient and unwieldy, which resulted in the production units being relatively small in size. Consequently, the flow rate was low, and its annual production was less than 10,000 tons. The UIP1500hdT ultrasonic processing system was used to process three scenarios. It was found that the third scenario (direct sale of crude glycerol without methanol recovery) had the lowest cost and the highest return on investment was 334%. Although there was no recovery of methanol and cleaning the ester layer caused some waste, this situation had the highest profit. The use of an ultrasonic treatment system not only reduced the fixed costs of the biodiesel distillation tower but also reduced utility costs, particularly by reducing the cooling water temperature of the cooler and the steam temperature of the reboiler [48]. It is evident that a number of challenges and impediments must be surmounted in order to realize the expansion and industrialization of biodiesel production. Table 5 showed the cost of producing one ton of biodiesel from different raw materials under different catalysts.

### 4.2. Environmental Analysis

Catalysts for biodiesel production from solid waste have good economic potential, but their impact on the environment cannot be ignored. The environmental (E) factor refers to the ratio of the total waste produced in the production process to the quality of the target product. It is usually used to evaluate the environmental sustainability of chemical processes, and the ideal E factor is zero. If the E value is higher, more waste is generated in the chemical process, and the more serious the harm to the environment. The E factor of the biodiesel process catalyzed by the BaAl_2_O_4_ catalyst prepared by the co-precipitation method using pure chemical reagents is as high as 3.85 [179]. High temperature calcination as a non-negligible factor needs to be considered. The parameter of environmental factor E obtained by Ribeiro et al. [26] using biochar to prepare a magnetic catalyst was 0.145, which is close to zero. Compared with the environmental factor of 0.162 obtained by Roy et al. [180], for the catalyst for potassium-promoted lanthanum oxide prepared by the sol–gel method, the environmental factor was reduced by 11.7%. It also shows that the use of solid waste to prepare catalysts is a sustainable and more ecological way.

In addition to environmental factors, life cycle assessment (LCA) can also be used to analyze the environmental impact of product preparation and consumption processes. LCA is a scientific method that considers all processes involved in the life cycle of a product, from raw material acquisition to product production, use, and disposal [181]. The assessment covers the entire biodiesel life cycle, starting from the collection and pretreatment of raw materials, continuing with the utilization of energy and chemicals in the biodiesel production stage, and concluding with greenhouse gases and particulate matter generated during the transportation and combustion of biodiesel. In the consideration of factors such as pollutants, potential health risks, and waste management, the goal was to minimize the environmental impact of the entire biodiesel life cycle and ensure the sustainable use of waste resources [182]. The life cycle assessment was divided into two parts. Firstly, the impact on the environment, human health, and resources was assessed. Secondly, the impact on human health, ecosystems, and resources was assessed. A life cycle assessment was conducted on a sulfonated magnetic biochar catalyst derived from palm kernel shells. The assessment index for global warming, acidification, and ecological toxicity was found to be higher than that of a life cycle assessment using a NaOH homogeneous catalyst. This was primarily attributable to the generation of specific hazards during the process of waste treatment [183]. Through endpoint indicators, it was found that the entire catalyst preparation process had the greatest impact on human health. This may be due to the use of volatile chemical reagents in the preparation process, which increases the emission of harmful gases. The preparation process can be optimized to reduce pollutant emissions.

### 4.3. Heterogeneity Analysis

By discussing the heterogeneity of solid waste, the development of waste resources from ‘usable’ to ‘usable’ is realized, which will reduce the burden of industrial development and realize the high-value utilization of waste. Compared with pure substances, solid waste as the main source of the catalyst has the characteristics of wide distribution and great variation. Therefore, the thermal Shelton filtration method is usually used to test the heterogeneity of solid waste-based catalysts to determine their renewability. Under the optimal reaction conditions for a certain time, the catalyst is separated and the biodiesel yield is detected. Subsequently, the reaction time is extended to measure the biodiesel yield [6,184,185]. The comparison of the two results showed that the negligible trace element dissolution in the catalyst proved that the catalyst was heterogeneous. Good heterogeneity promotes the construction of the active sites of the catalyst and prolongs its cycle life to achieve higher reproducibility. After the reaction, the catalyst was centrifuged, washed and dried [27,109]. Macromolecular organic matter can reduce the pore blockage of the catalyst and improve its reproducibility. The magnetic catalyst prepared by Wang et al. [89] was not only beneficial to separation, but also can achieve 12 stable cycles without subsequent treatment, which provides a good reference for the later study of catalyst heterogeneity.

### 4.4. Preparation Method Analysis

Using solid waste to prepare catalysts to produce biodiesel is not only conducted to deal with solid waste, but also to fully consider the catalytic activity of solid waste-based catalysts and the method of preparing biodiesel. Common catalyst preparation methods include acid-base modification, impregnation, co-precipitation, calcination, etc. Due to the heterogeneity of solid waste itself, the direct use of catalytic biodiesel has not achieved good results. The purification of crude biodiesel and the treatment of unreacted solvents affect its industrial development. The acid-base modification of solid waste is conducive to the extraction of elements or substances with catalytic potential as active substances or carriers for catalysts. At the same time, the structure of the catalyst can also be changed. For example, plant solid waste can be sulfonated by sulfuric acid to form biochar, changing its original loose structure. However, the use of acids and alkali leaches some heavy metal elements in solid waste or elements harmful to the catalyst. Therefore, when we choose the modifier, we should consider the influence of different elements on the catalytic reaction and the environmental impact following the environment after the end of the difference. The impregnation method involves introducing active substances (metal ions or metal oxides) into the catalyst carrier. These impregnated metal ions or oxides can not only enhance the acid-base sites of the catalyst, but also modify its morphology of the catalyst by physical and chemical adsorption to stabilize its structure. However, the distribution uniformity and the introduction of impurities after impregnation affect the activity and selectivity of the catalyst. The co-precipitation method addresses some shortcomings of the impregnation method. By mixing a solution containing a variety of metals with the precipitant, the metal ions are precipitated. In the process of precipitation, each component is evenly distributed, and the particle size or crystal form of the precipitate can be completed by adjusting the reaction conditions. However, due to improper pH control, component deviation will occur, and even agglomeration will occur to reduce the specific surface area. As the most common catalyst preparation method, high temperature calcination can change the active component and structure of the catalyst, change the pore distribution and specific surface of the catalyst, and remove the impurities and some organic solvents of the catalyst. High temperature calcination needs to consume a lot of energy and is not environmentally friendly. Even if the temperature is too high, the catalyst sintering will reduce the catalytic activity.

Hydrothermal synthesis and nano-synthesis technology are new technologies for preparing catalysts. Hydrothermal synthesis technology is a catalyst that uses water as a solvent to dissolve and crystallize the reactants under high temperature and high pressure conditions to form a stable structure. Hydrothermal synthesis technology does not require additional template preparation. It can adjust the relevant reaction parameters to control the morphology and pore structure of the catalyst particles and optimize the mass transfer performance. The hydrothermal synthesis reaction has a long reaction cycle and high energy consumption, which is not suitable for large-scale production. Nano-synthesis technology refers to the preparation of nano-structured catalysts by the sol–gel method, co-precipitation method, microemulsion method, and other specific methods. The nanoscale structure produces more active sites, optimized the adsorption effect of the reactants, and even combined the advantages of multiple components to achieve synergistic transformation. Nanocatalysts may undergo structural damage and even some agglomeration in a high temperature and strong corrosion environment. Some of the current preparation methods are complicated and limit their industrial production. By summarizing the advantages and disadvantages of the catalyst preparation methods, relevant methods for the preparation of more waste-based catalysts to improve the catalytic performance of the catalyst are provided.

## 5. Conclusions and Future Perspectives

Although solid waste-based catalysts possess the catalytic activity of traditional heterogeneous catalysts, their catalytic stability still has certain shortcomings. In particular, during the activation process, the material tends to agglomerate or form lumps, resulting in the poor adhesion of the active substance. The application of nanotechnology greatly improves the structure of the catalyst, expands its pore size, and demonstrates its excellent catalytic performance. The doping of magnetic material enables the catalyst to be directly recycled, reducing the tedious process of filtration. The price of biodiesel produced by using solid waste as a catalyst is lower than that of petroleum diesel. If waste oil is used as raw material, the cost can be further reduced, demonstrating significant commercial development potential. For the long-term development of biodiesel, the high activity of the catalyst, the cost of the complex preparation process, and the environmental impact of production or deactivation are the main challenges. This review emphasizes the efficient utilization of solid waste-based catalysts in biodiesel production and explores potential solutions. This paves the way for the future commercial and sustainable use of catalysts derived from solid waste.

Looking to the future, we will continue to pay attention to solid waste with the potential to develop catalysts in various industries and the latest processes of using solid waste to prepare catalysts. The synthesis materials, preparation process, and optimization methods of the catalysts were mainly studied to promote their sustainable development. The application of nanostructures and metal–organic frameworks will further improve the catalytic efficiency and thermal stability, and solve the problems of catalyst deactivation and low cycle efficiency. With the high demands for process simplification and catalytic costs, the development of catalysts with dual functions of magnetic properties and acid-base properties has a positive impact on catalyst recovery and the catalytic cracking of raw oil with high acid value. The regeneration and treatment of deactivated catalysts is also a problem that cannot be ignored in the production of biodiesel. The heavy metal elements in solid waste will always exist in the catalyst, and improper treatment still causes serious environmental pollution. Future research aims to improve efficiency, promote the bio-recycling economy and reduce environmental pollution to improve its own competitiveness. Waste-based catalysts can reuse solid waste and promote the sustainable use of solid waste to improve resource efficiency and environmental protection. In general, heterogeneous catalysts derived from solid waste provide a reliable solution for waste-management issues. They offer an effective and environmentally friendly option for biodiesel production.

## Figures and Tables

**Table 1:** Preparation of biodiesel using different agricultural wastes as catalysts.

No.	Catalyst Source	Feedstock	Catalyst	Optimum Conditions				Yield (%)	Citation
				A	B	C	D		
1	Rice husk	UCO	MoO_3_/RHA-CoFe_2_O_4_	20:1	6	240	160	94.60	[24]
2	Betel leaf	*Soybean oil*, *jatropha oil*, and *pongamia oil*	Biochar/K_2_CO_3_	9:1	15	201	65	96.57	[25]
3	Murici seeds	*Soybean oil*	Biochar/CoFe_2_O_4_	17:1	7	84	90	97.11	[26]
4	Banana peel	*Safflower oil*	C/Zn	22.4:1	6.63	53.55	85	97.12	[27]
5	Jackfruit peel	UCO	K/Ca/Mg	9:1	12	105	65	97.42	[28]
6	Rice husk	*Dairy waste oil*	CuO/RHA	11.12:1	2.76	171	62.36	97.42	[29]
7	Banana peel	*Palm oil*	K_2_O/Na_2_O	6:1	2	90	65	98.06	[30]
8	Banana peel, stem, rhizome	*Neem oil*	K_2_O/CaO	9:1	5	10	65	98.27	[21]
9	Sorghum-sugarcane bagasse	*Pork fat oil*	K/Ca/Mg	8.57:1	3.15	69.96	79.93	98.52	[31]
10	Orange peel—Banana peel	*Flaxseed oil*	CaO	10:1	3	50	70	98.78	[23]
11	Palm kernel shell	Used *palm oil*	Na_2_O/SiO_2_	15:1	6	90	50	99.01	[32]
12	Corn pod	*Papaya oil*	CaO/K_2_O	5.99:1	3.96	72.42	70	99.06	[33]
13	Rice husk	*Oleic acid*	Biochar/H_2_SO_4_	24:1	8	60	80	99.60	[34]
14	Bamboo—Coconut shell	*Oleic acid*	Biochar/H_2_SO_4_	9:1	3	40	80	99.60	[35]

A: molar ratio (methanol:oil); B: catalyst loading (wt%); C: time (min); D: temperature (°C).

**Table 2:** Preparation of biodiesel using metallurgical waste as a catalyst.

No.	Catalyst Source	Feedstock	Catalyst	Optimum Conditions				Yield (%)	Citation
				A	B	C	D		
1	Blast furnace dust	*Palm oil*	Fe/CaO	15.24:1	7.96	120	148.95	87.67	[80]
2	Red mud—Brazilian berry seeds	*Oleic acid*	Fe/H_2_SO_4_	12:1	5	60	100	88	[81]
	Blast furnace slag	UCO	SFCA	20:1	20	720	60	90.77	[82]
4	Blast furnace slag	UCO	K_2_SiO_3_	15:1	5	180	60	93.15	[83]
5	Red mud	*Soybean oil*	Ca/Ti/K	24:1	4	180	65	94	[84]
6	Red mud	*Castor oil*	RM/K	18:1	5	150	65	95	[85]
7	Aluminum industry scrap	UCO	CaO/Al_2_O_3_	7:1	3	180	45	95	[86]
8	Red mud	*Castor oil*	RM/Li	15:1	5	150	65	96.32	[87]
9	Aluminum industry scrap	UCO	KAlO	9:1	5.8	120	25	98.7	[88]
10	Electric furnace dust—reed straw	*Soybean oil*	Fe/Na_2_CO_3_	14.06:1	7.75	249.6	74.15	99.89	[89]
11	Steel ladle furnace slag—kaolin	*Soybean oil*	CaSiO_3_	7.5:1	3	240	60		[90]

A: molar ratio (methanol:oil); B: catalyst loading (wt%); C: time (min); D: temperature (°C).

**Table 3:** Biodiesel production by transesterification using various shell-derived solid base catalysts and different feedstocks.

No.	Catalyst Source	Feedstock	Catalyst	Optimum Conditions				Yield (%)	Citation
				A	B	C	D		
1	Eggshell	*Chicken fat oil*	CaO/Fe	15:1	3	300	65	83	[129]
2	Eggshell	UCO	CaSO_4_/Fe_2_O_3_	5:1	15	201	65	89.45	[130]
3	Eggshell	UCO	CaO	22.5:1	4	45	70	91	[131]
4	Eggshell	UCO	CaO-ZSM/Fe	5:1	3.5	330	65	91	[132]
5	Eggshell	UCO	CaO/MeOH	10:1	1.5	90	60	93.1	[133]
6	Eggshell-Zeolite	UCO	CaO	9.7:1	2.1	238.8	69.1	93.7	[134]
7	Eggshell	*Rapeseed oil*	CaO/Na-K	9:1	3	180	50	97.6	[135]
8	Eggshell	*Rubber seed oil*	CaO	9:1	5	240	60	97.84	[136]
9	Eggshell	*P. pinnata oil*	CaO/Fe	12:1	2	120	65	98	[137]
10	Eggshell	*Castor bean seed oil*	CaO	8.44:1	2.78	108	60	98.31	[138]
11	Eggshell—Poplar Leaf	*Sheep fat oil*	CaO-SrO/C	8:1	6	90	65	98.83	[139]
12	Eggshell	*Argemone mexicana oil*	CaO	9.7:1	3.05	180	60	99.07	[140]
13	Eggshells—chicken bones	UCO	CaO-HAp/MnFe-K	15.24:1	2.97	175.72	67.72	99.1	[141]

A: molar ratio (methanol:oil); B: catalyst loading (wt%); C: time (min); D: temperature (°C).

**Table 5:** Economic analysis of biodiesel production from solid waste.

No.	Feedstock	Catalyst	Price (T/USD)	Citation
1	UCO	Used alkaline batteries	579	[166]
2	*Soybean oil*	Eggshell/MOF@ZnCo-LDH	420	[8]
3	*Sheep fat oil*	Poplar leaves/eggshells	522	[139]
4	*Palm fatty acid distillate*	Onion peel/sulfuric acid	114	[167]
5	*Safflower oil*	Banana peel/sulfuric acid	814	[27]
6	*Kanuga oil*	Coconut shell/sulfuric acid	833	[168]
7	UCO	Rubber seed shells/eggshells	370	[169]
8	UCO	Orange peel	1120	[170]
9	UCO	Eggshell/ZnFe_2_O_4_	584	[171]
10	UCO	Corn cob/KOH	776	[48]
11	*Castor oil*	Red mud/K_2_CO_3_	718	[85]
12	*Neem oil*	Animal skeleton	1060	[165]
13	FFA	Al_2_O_3_/HSiW	2240	[172]
14	*Soybean oil*	CaO/ZnO	1110	[173]
15	OA	FPW-HK	593	[174]
16	*Soybean oil*	Mo/Ce/H-TiO	1090	[175]
17	*Castor oil*	NaOH	1496	[176]
18	*Soybean oil*	CaO	1294	[119]
19	UCO	SrO–ZnO/MOF	710	[177]
20	*P. pinnata oil*	Fe3O4/SiO_2_/PAIL	980	[178]

## Data Availability

No new data were created or analyzed in this study. Data sharing is not applicable.

## Abbreviations

The following abbreviations are used in this manuscript:

WOO	World Oil Outlook
UNEP	United Nations Environment Programme
EDS	Energy Dispersive Spectroscopy
FAME	Fatty Acid Methyl Esters
FESEM	field emission scanning electron microscope
FI-IR	fourier transform infrared spectrometer
XPS	X-ray photoelectron spectroscopy
EDX	Energy Dispersive X-Ray Spectroscopy
TGA	Thermal Gravimetric Analyzer
BET	Brunauer–Emmett–Teller
ICP	Inductively Coupled Plasma
CO_2_-TPD	Carbon Dioxide Temperature Programmed Desorption
GC-MS	Gas chromatography mass spectrometry
HAP	Hydroxyapatite
β-TCP	β-tricalcium phosphate
RSM	Response Surface Methodology
FCCD	face centered central composite design
MOF	metal–organic framework
TP	Tetra Pak
PWC	plastic waste char
UCO	Used Cooking Oil
ASTM	American Society of Testing Materials
IRI	infrared irradiation
LCA	life cycle assessment
NPV	Net Present Value
